# Clinical outcomes and direct cost analysis of rotator cuff repair surgery

**DOI:** 10.31744/einstein_journal/2024GS0473

**Published:** 2024-08-05

**Authors:** Rafael Pierami, Eliane Antonioli, Isabela Queiros Castro, Paula Fairbanks, Felipe Giorgi Manente, Mario Lenza

**Affiliations:** 1 Hospital Israelita Albert Einstein São Paulo SP Brazil Hospital Israelita Albert Einstein, São Paulo, SP, Brazil.; 2 Hospital Alvorada Moema São Paulo SP Brazil Hospital Alvorada Moema, São Paulo, SP, Brazil.; 3 Faculdade Israelita de Ciências da Saúde Albert Einstein Hospital Israelita Albert Einstein São Paulo SP Brazil Faculdade Israelita de Ciências da Saúde Albert Einstein, Hospital Israelita Albert Einstein, São Paulo, SP, Brazil.

**Keywords:** Rotator cuff, Shoulder pain, Cost-effectiveness analysis, Costs and cost analysis, Health care costs, Treatment outcomes, Arthroscopy, Orthopedic procedures

## Abstract

**Objective:**

The purpose of this study was to evaluate the clinical and functional outcomes in patients who underwent surgical treatment for rotator cuff tears using open and arthroscopic techniques, and to evaluate the direct costs involved.

**Methods:**

Retrospective cohort study with analysis of the data of patients who were referred to two private hospitals in Sao Paulo, Brazil for surgical repair of the rotator cuff from January 2018 to September 2019. Clinical outcomes were assessed using functional scores (SPADI and QuickDASH) and a quality of life questionnaire (EuroQoL). Procedure costs were calculated relative to each hospital’s costliest procedure.

**Results:**

Data from 362 patients were analyzed. The mean patient age was 57 years (SD= 10.46), with a slight male predominance (53.9%). Arthroscopic procedures were more common than open procedures (95.6% *versus* 4.4%). Significant clinical improvement was reported in 84.8% of the patients. The factors associated with increased surgery costs were arthroscopic technique (increase of 29.2%), age (increase of 0.6% per year), and length of stay (increase of 18.9% per day of hospitalization).

**Conclusion:**

Rotator cuff repair surgery is a highly effective procedure, associated with favorable clinical outcomes and improvement in life quality, and low rates of complications. Arthroscopic surgery tends to be costlier than open surgery.

## Graphical abstract

**Figure:**
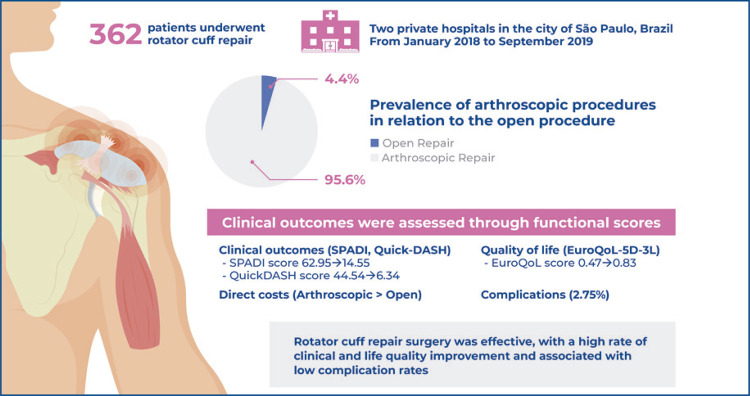

## INTRODUCTION

Shoulder pain is associated with a set of dysfunctions that comprise the second most common musculoskeletal complaint after lumbar pathologies.^(1-3)^ Approximately 4.5 million medical consultations are performed annually in the United States of America due to this complaint.^(1,2)^ Rotator cuff syndrome is considered to be the main cause of shoulder disability.^(2)^The syndrome encompasses a wide range of alterations, from tendon degeneration, such as tendinosis or tendinopathy, to tears, which may be partial or complete.^(2)^Its multifactorial etiology is associated with age, trauma, and other elements intrinsic or extrinsic to the patient.^(1,3,4)^Diagnosis is based on clinical history, physical examination, and imaging results. Magnetic resonance imaging (MRI) is considered the gold standard.^(5)^

Traditionally, surgery has been indicated for symptomatic patients with total or partial high-grade tears, that is, tears that involve 50% or more of the tendon thickness, and for patients who do not respond to or tolerate non-surgical treatment, including physical therapy, changes in daily activities, and medications. The two commonly performed surgical techniques for^(1)^ rotator cuff repair-open and arthroscopic-have a high success rate, with no evidence of superiority between them.^(1,4,6)^

A study published in 2019 by the American Academy of Orthopaedic Surgeons (AAOS) reported that approximately 250,000 rotator cuff repair surgeries are performed annually in the United States, with an estimated cost of $3.4 billion. Moreover, surgery rates are rising significantly,^(1)^and this trend is expected to continue over the next years. A 238% increase has been reported in the number of such surgeries in the Brazilian Public Health System (SUS - *Sistema Único de Saúde*) between 2003 and 2015.^(7)^With the expected aging of the population and inversion of the Brazilian age pyramid, rotator cuff tears are projected to increase exponentially in the coming decades, making the treatment of these lesions a real public health concern.

## OBJECTIVE

In view of the growing importance of this pathology in the Brazilian population and the associated economic impact, the present study sought to evaluate the clinical outcomes 12 months after rotator cuff surgical repair and the direct costs involved in this procedure by analyzing data from the two surgical techniques in two private hospitals in the city of São Paulo.

## METHODS

We retrospectively analyzed data from patients who underwent rotator cuff repair surgery in two private hospitals in the city of São Paulo from January, 2018 to September, 2019. The study was approved by the Research Ethics Committee of *Hospital Israelita Albert Einstein*, under CAAE: 19182619.3.1001.0071; #4.333.262.

Data from 725 rotator cuff repair surgeries were accessed, and 363 were excluded from the analysis due to missing values. The following data were collected and analyzed: age, sex, laterality, type of surgery (open or arthroscopic), length of hospitalization, complications, functional scores, and quality of life scores. Data included the preoperative period and 12 months after surgery. We also obtained data regarding the direct cost of the procedure, that is, the costs associated with the period of hospitalization and directly related to the surgical procedure, such as hospitalization length, time of use of the operating room, list of medications, and surgical materials and implants used during surgery. Information on the effective direct cost of surgery in Reais was not available for the two hospitals because one provided the direct cost of hospitalization in relation to the highest cost procedure; information was thus available of the proportional cost (percentage) instead of the absolute value. To standardize the data, we transformed absolute costs into relative costs and calculated the proportional cost for each patient in relation to the costliest procedure in each hospital.

The hospitals involved in this study use different functional scores to determine the effectiveness of rotator cuff repair surgery: Shoulder Pain and Disability (SPADI) and QuickDASH. The SPADI functional score consists of 13 items across pain and function domains, with each item scored from 0 to 10. The score is interpreted as a percentage on a scale from 0 to 100, with higher scores indicating worse function.^(8)^ QuickDASH uses 11 items to measure function and symptoms of upper limb diseases. Each item is scored from 1 to 5 and the sum of these values is transformed into a percentage from 0 to 100, with higher values indicating worse upper limb function.^(9)^As the hospitals used different functionality scores, to assess functional outcomes, significant clinical improvement was classified according to the minimal clinically important difference (MCID) of the respective patient scores. Accordingly, a difference ≥18 points in SPADI^(10)^ or 20 points in QuickDASH^(11)^signified clinical improvement.

The EuroQol^(12)^ tool is composed of questions that evaluate five dimensions of quality of life: mobility, self-care, daily activities, pain/discomfort, and anxiety/depression. The results range from 0 (worst health status) to 100 (best health).

### Statistical analysis

The data are presented as the mean ± standard deviation (SD), minimum and maximum, median, or quartiles for quantitative variables, and by absolute and relative frequencies for qualitative variables. The normality of the data was verified using the Shapiro-Wilk test, bloxplots, histograms, and quantile comparison charts.

The costs were analyzed as relative values in relation to the costliest procedure in each hospital. To analyze the factors associated with the proportional cost of the procedures, we used Gamma regression from Generalized Linear Models (GLM). Simple and multiple models were adjusted, considering the characteristics of the patients and clinical evolution 12 months after surgery. The results are presented as mean ratios, 95% confidence intervals, and p-values.

For the analysis of factors associated with significant clinical improvement, we used simple and multiple logistic models, considering sex, smoking status, length of hospital stay, type of surgery, occurrence of complications, and variation in the measure of quality of life (assessed using the EuroQoL score) as explanatory variables. The results are presented as odds ratios, 95% confidence intervals, and p values.

The analyses were performed using the SPSS v.26.0^13^ software and a 5% significance level was adopted.

## RESULTS

Table 1 presents the data of patients who underwent rotator cuff repair surgery, according to surgery type. Data from 362 patients in the two hospitals were analyzed. Mean age was 57.08 years in arthroscopic surgery (SD = 10.59; minimum = 24 years and maximum = 83 years) and 59.75 in open surgery (SD = 10.31; minimum = 44 years and maximum = 73 years). We observed a higher proportion of male patients (n= 195; 53.9%). Regarding surgery type, 346 patients (95.6%) had arthroscopic surgery and 16 (4.4%) had open surgery. The median length of stay was 1.0 days (IIQ= 0.92-1.17). Complications occurred in 2.8% of patients (n=10), all in the arthroscopic procedure group.

**Table 1:** Patient characteristics

Number of patients	Arthroscopic surgery n=346	Open surgery n=16
Institution (n=362), n (%)		
Hospital A	206 (56.90)	12 (3.30)
Hospital B	140 (38.70)	4 (1.10)
Age (n=362)		
Mean (SD)	57.08 (10.59)	59.75 (10.31)
Min–Max	24–83	44–73
Sex (n=362), n (%)		
Male	188 (51.90)	7 (1.90)
Female	158 (43.60)	9 (2.50)
Length of hospitalization (n=362)		
Median (IIQ)	1 (0.23)	1 (0.34)
Min–Max	0.93–1.16	0.84–1.19
Complications (n=355), n (%)		
No	330 (93)	15 (4.20)
Yes	10 (2.80)	0 (0)

SD: standard deviation; IIQ: interquartile range.

### Functional and Quality of Life Scores

Clinical improvement was assessed based on the MCID values from SPADI and QuickDASH, 12 months after surgery compared to the previous time point. Significant clinical improvement was noted in 307 patients (84.8%). The effect was observed in most patients who were treated with open repair (15 of 16; 93.75%) and in 292 of the 346 (84.4%) patients who underwent arthroscopic repair. Quality of life, measured using EuroQoL-5D-3L, generally improved after 12 months, with a mean increase of 0.38 for the open technique and 0.49 for the arthroscopic technique (Table 2).

**Table 2:** Clinical outcomes

Outcome	Time/Clinical improvement	Arthroscopic surgery	Open surgery
EuroQoL-5D-3L, (Median) (%)	Baseline	0.51 (-0.60–1.00)	0.42 (0.31–0.60)
	12 months	1.00 (0.69-1.00)	0.80 (0.69-1.00)
QuickDASH (n=144)	Baseline	44.31 (29.54–61.36)	52.27 (22.77–60.22)
(Median) (%)	12 months	0.10 (0.10–6.81)	4.59 (0.10–27.27)
SPADI (n=218)	Baseline	0.69 (0.49–0.80)	0.69 (0.52–0.80)
(Median) (%)	12 months	0.07 (0.00–0.19)	0.06 (0.00–0.30)
Clinical improvement, n (%)	No Improvement	54 (14.90)	1 (0.30)
	Improvement	292 (80.70)	15 (4.10)

In the secondary analysis, we sought to identify the factors that may be associated with surgical success. To this end, we performed logistic regression and analysis using simple and multiple models to assess potential associations between clinical improvement and several variables. We observed that female sex (p=0.007) was positively associated with clinical improvement, as did absence of acute complications (p=0.013) and improvement in quality of life (p=0.041) (Table 3). Women were almost threefold more likely to report significant clinical improvement compared to men (composite reliability [CR] = 2.956; p=0.007). Absence of complications during hospitalization increased the likelihood of significant clinical improvement by eight times (CR = 8.158; p=0.013). Lastly, improvement in the EuroQol score increased the likelihood of significant clinical improvement, with a 2.3% increase for each unit of difference between the time points assessed (CR = 1.023; p=0.041).

**Table 3:** Factors associated with clinical improvement

	Simple model		Multiple model	

	OR	p value	OR	p value
	
	(95%CI)		(95%CI)	
Sex				
Female	2.359 (1.265–4.401)	0.007	2.956 (1.35–6.477)	0.01
Male		-		-
Smoking				
Yes	1.328 (0.38–4.635)	0.657	1.984 (0.437–9.004)	0.38
No		-		-
Age	1.009 (0.982–1.036)	0.525	1.004 (0.972–1.037)	0.79
Length of hospitalization	0.999 (0.639–1.562)	0.997	1.013 (0.527–1.947)	0.97
Surgery method				
Arthroscopic	0.36 (0.047–2.786)	0.328	1.155 (0.132–10.084)	0.9
Open		-		-
Complication				
No	9.75 (2.65–35.872)	<0.01	8.158 (1.57–42.399)	0.01
Yes		-		-
EuroQoL (Baseline-6 months)	1.03 (1.011–1.05)	0.002	1.023 (1.001–1.047)	0.04

OR: odds ratio; 95%CI: 95% confidence interval.

**Figure 1:**
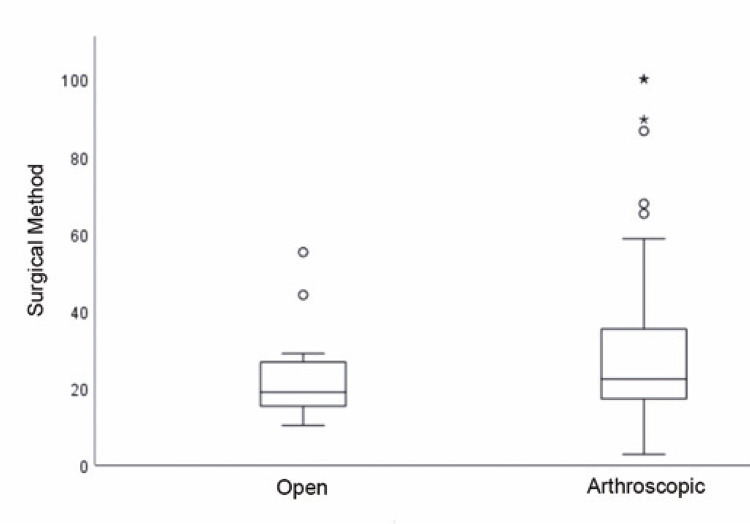
Cost comparison (in proportion) by type of repair surgery

The costs of the procedures were estimated as percentages relative to the costliest procedure in each hospital. The average proportional cost was 26.97% (27.16% *versus* 22.85% for arthroscopic and open surgery, respectively), with a variation between 2.83% and 100% (Figure 1).

To identify any associations between costs with characteristics related to hospitalization and patient evolution, we used gamma regression models. Simple association models showed that the factors associated with cost increases were occurrence of acute complications (p=0.002), age (p<0.001), and length of stay (p<0.001). In the multiple model, the factors that remained associated with cost were patient age, with an expected increase of 0.6% in proportional cost for each year of age (RM = 1.006, p=0.004), and length of stay, with each additional day of hospitalization resulting in an 18.9% increase in proportional cost (RM = 1.189, p<0.001). In contrast, and unexpectedly, absence of acute complications was associated with an estimated 40.9% increase in the direct cost of the procedure (RM = 1.409; p=0.016) (Table 4).

**Table 4:** Association between proportional cost and characteristics: gamma regression models

Variables	Simple model		Multiple model	

	Mean ratio	p value	Mean ratio	p value
	
	(95%CI)		(95%CI)	
Sex	1.072 (0.972–1.181)	0.164	1.007 (0.918–1.106)	0.879
Female	Reference		Reference	
Male				
Surgical method	1.89 (0.942–1.499)	0.145	1.292 (1.035–1.614)	0.024
Arthroscopic	Reference		Reference	
Open				
Occurrence of acute complications	1.590 (1.190–2.124)	0.002	1.409 (1.065–1.865)	0.016
No	Reference		Reference	
Yes				
Significant improvement	0.950 (0.831–1.087)	0.459	1.001 (0.871–1.150)	0.985
No	Reference		Reference	
Yes				
Length of hospitalization (days)	1.224 (1.147–1.306)	<0.001	1.189 (1.121–1.261)	<0.001
EuroQoL difference (baseline and 1 year)	1.350 (1.125–1.619)	0.001	1.152 (0.981–1.354)	0.084
Age	1.008 (1.004–1.013)	<0.001	1.006 (1.002–1.010)	0.004

## DISCUSSION

Rotator cuff repair is the most common shoulder surgery.^(1,14-16)^As reported by Malavolta et al.,^(7)^the number of procedures performed in Brazil between 2003 and 2015 has increased significantly and is expected to increase considering the estimated aging of the Brazilian population. Thus, information on the effectiveness of this procedure, involved costs, and factors related to surgical outcomes is essential.

Yamaguchi et al.^(17,18)^ analyzed data from 588 consecutive patients with rotator cuff tendon ruptures and showed a correlation between unilateral rupture with age of 58.7 years or older. Likewise, Teunis et al.^(19)^ demonstrated a correlation between rotator cuff pathologies and age, even in asymptomatic patients, similar to the findings of other epidemiological and review studies. We found that the mean age of patients who underwent arthroscopic or open surgery for rotator cuff repair was 57.08 and 59.75 years, respectively, which is in agreement with the literature.

Surgical treatment of rotator cuff tears offers excellent clinical results, with a success rate of approximately 90%.^(1,4,20)^ Moreover, the procedure has low complication rates, which is generally associated with limited clinical repercussions and minimal influence on treatment outcomes. Our study corroborates previous findings, demonstrating a clinical functional improvement rate in 85% of the patients and a rate of 5.6% for complications (10 of 355).

The development of the arthroscopic technique and the evolution of arthroscopic surgical devices has transformed how rotator cuff repair surgery is taught and performed. Possible advantages of the arthroscopic procedure include smaller incision sizes and lesser need for muscle dissection, especially of the deltoid muscle; consequently, postoperative pain, muscle damage, and need for opioid analgesics are minimized. For these reasons and the great enthusiasm for new devices and arthroscopic techniques, an increasing shift from open repair to arthroscopic repair for these tears has been noted.^(21,22)^ In our cohort, 95% of the rotator cuff repair surgeries were performed using the arthroscopic technique, corroborating previous evidence. Despite the large disparity in the number of arthroscopic and open procedures, both showed similar rates of significant clinical improvement.

Considering the amount of materials and equipment required to perform the arthroscopic procedure and previous descriptions in the literature, the higher cost related to this procedure, when compared to that of the open technique, may be justified.^(20,23-25)^

Cost increases in rotator cuff repair surgeries are mainly related to length of hospital stay, surgery time, number and type of anchors used, additional surgical procedures such as acromioplasty, resection of the lateral portion of the clavicle, or joint debridement, and comorbidities.^(26-28)^ In the present study, through association analysis, we identified that age, length of hospitalization, and, surprisingly, lack of acute complications are factors that influence the cost of the procedure. This paradox may be explained by the large disparity in the number of arthroscopic and open surgeries performed. A robust interpretation of this analysis is limited considering that all complications were described in the arthroscopic surgery group, a result that was probably associated with the number of procedures in each type of technique.

The present study had some limitations that are worth mentioning. First, it was based on retrospective data, which may have contained inaccurate records. However, we believe that the selected data were accurate because they were supplied by the teams that manage and organize all orthopedic surgical cases at the institutions. Second, the number of open procedures was significantly lower than that of arthroscopic procedures, which limited the interpretation of the analyses.

## CONCLUSION

The analyses presented here allow us to conclude that rotator cuff repair surgery, whether performed using the open or the arthroscopic technique, results in significant clinical improvement for most patients and is thus effective. Moreover, the procedure has a low complication rate. Factors associated with poor clinical outcomes were male sex and occurrence of complications.
